# Signal intensity on fluid-attenuated inversion recovery images of condylar marrow changes correspond with slight pain in patients with temporomandibular joint disorders

**DOI:** 10.1007/s11282-014-0165-5

**Published:** 2014-02-16

**Authors:** Sayaka Kodama, Mika Otonari-Yamamoto, Tsukasa Sano, Junichirou Sakamoto, Kenichi Imoto, Mamoru Wakoh

**Affiliations:** 1 Department of Physiology, Tokyo Dental College, Misakichyo 2-1-14, Chiyoda-ku, Tokyo, 101-0061 Japan; 2 Department of Oral and Maxillofacial Radiology, Tokyo Dental College, Tokyo, Japan; 3 Division of Radiology, Department of Oral Diagnostic Sciences, Showa University School of Dentistry, Tokyo, Japan; 4 Oral and Maxillofacial Radiology, Graduate School, Tokyo Medical and Dental University, Tokyo, Japan

**Keywords:** Bone marrow, Fluid-attenuated inversion recovery (FLAIR), Magnetic resonance imaging (MRI), Temporomandibular joint (TMJ)

## Abstract

**Objectives:**

Edema and necrosis of the temporomandibular joint (TMJ) have been described in terms of bone marrow signal abnormalities in magnetic resonance imaging (MRI). However, painful joints often show no such signaling abnormalities, making the diagnosis of TMJ disorders difficult in the clinical setting. An association has been suggested between TMJ bone marrow change and TMJ pain, but even when such change results in slight pain, it may be too slight to be visually apparent on MR images. We hypothesized that fluid-attenuated inversion recovery (FLAIR) can be used to detect such minimal changes. The purpose of this study was to determine whether there is an association between signal intensity on FLAIR images and pain in the TMJ.

**Methods:**

The study included 85 TMJs in 45 patients referred to our department for MRI. The signal intensity on FLAIR images was measured. Pain was evaluated based on the visual analog scale. An unpaired *t* test and Pearson’s product-moment correlation coefficient were used for the statistical analysis. A *p* value of <0.05 was considered statistically significant.

**Results:**

Signal intensity on the FLAIR images was significantly higher in painful than in nonpainful TMJs, although a significant correlation was not observed between the signal intensity and the pain score.

**Conclusions:**

The results of this study suggest an association between abnormalities in the marrow of the mandibular condyle and pain. They also indicate that FLAIR imaging is a useful tool in the clinical diagnosis of painful TMJs.

## Introduction

Pain is a primary concern in patients with disorders of the temporomandibular joint (TMJ) [1, 2]. The precise cause of this pain, however, remains to be clarified [3, 4]. Pathological change in the various anatomical structures of the TMJ has been suggested as one possible explanation. Edema and necrosis reportedly result in abnormalities in signals generated by the bone marrow in magnetic resonance imaging (MRI) of the TMJ [5–11]. Larheim et al. [8] found that such signaling abnormalities correlated with histological evidence of pathological change in the bone marrow as evidenced by core biopsy. Marrow edema has also been suggested to be a precursor of osteonecrotic development in the condyle. Sano et al. [9] showed that pain was markedly greater in joints with bone marrow change, including the mandibular condyle. A higher degree of pain was observed in joints with marrow edema than in those with osteonecrosis [12]. Edema has therefore been suggested to be a factor involved in the etiology of TMJ pain [9, 11].

MRI is generally performed to obtain proton density- and T2-weighted or T1- and T2-weighted images in patients with disorders of the TMJ. Such images allow for the detection of typical marrow abnormalities such as edema and osteonecrosis in the mandibular condyle. In the clinical setting, however, painful joints showing neither typical bone marrow changes nor other abnormalities are sometimes encountered when attempting to diagnose diseases of the TMJ based on MRI. If there is an association between change in the bone marrow and pain in the TMJ, it is possible that pain may occur even when such change is too small to be visually apparent on proton density- or T2-weighted images.

Imoto et al. [13] used fluid-attenuated inversion recovery (FLAIR)-sequence MRI to identify the biochemical elements of joint effusion, indicating the usefulness of this technique as a noninvasive diagnostic tool. We hypothesized that this technique can also be used to detect minimal changes in the bone marrow of the mandibular condyle. Accordingly, the purpose of this study was to investigate the association between signal intensity on FLAIR images and pain in the TMJ.

## Materials and methods

This study investigated 108 joints in 54 consecutive patients with symptoms of TMJ dysfunction. All patients were referred to our department at Tokyo Dental College Chiba Hospital for MRI in 2009. Informed consent was obtained from all patients, and the study protocol was approved by the ethical review board of our institution (No. 347). Twenty-three joints in 15 of these patients were excluded from the study as follows: 8 joints in 4 patients referred for MRI after surgery for jaw deformity; 7 joints in 6 patients with osseous change [7]; 3 joints in 2 patients with visually apparent marrow change on MR images; 2 joints in 1 patient because of potential difficulty in placing the regions of interest (ROI) because of body movement artifact; and 2 joints in 1 patient because of the absence of a visual analog scale (VAS) score. Moreover, an additional joint in one patient under 15 years of age was excluded in accordance with the Guidelines on the Treatment of TMJ Disorders established by the Japanese Society for the Temporomandibular Joint in 2001 [14]. Therefore, a total of 85 joints in 45 patients (32 women and 13 men) with suspected temporomandibular joint disorders other than osteoarthritis were included in the final analysis. The mean age of the patients was 34.3 years (range 15–82 years). Mental disorders or related histories were not observed in any patients.

Immediately prior to MRI, the patients rated their degree of pain upon occlusion using a VAS in which the maximum and minimum scores were 100 and 0, respectively. The degree of pain was scored separately for the left and right joints. The joints were categorized into a painful and a nonpainful group based on the median score, which was 2. The painful group included the joints with a VAS score of >2, and the nonpainful group included the joints with VAS score of <2. Four patients had both painful and nonpainful joints; each joint was categorized into the painful and nonpainful group, respectively.

MRI was performed using a 1.5 T MRI system (Magnetom Symphony, Maestro Class; Siemens, Erlangen, Germany) and a double-loop array of coils for the TMJ. Corrected sagittal images of the fast-spin echo sequence with FLAIR were obtained in the closed-mouth position (Table 1).

**Table 1:** FLAIR imaging parameters

Inversion time (ms)	2500
Repetition time (ms)	9000
Effective echo time (ms)	122
Echo train length	21
Field of view (mm)	150 × 150
Section thickness (mm)	3
Slice gap (mm)	0.3
Matrix	256 × 256

**Fig. 1:**
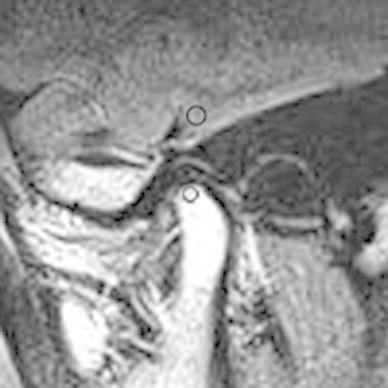
Measurement of region of interest (ROI) on corrected sagittal FLAIR images in closed-mouth position. The *lower circle* represents a 4.5-mm^2^ ROI in the bone marrow of the mandibular condyle; the *upper circle* represents a 5.9-mm^2^ ROI in the gray matter. Both were placed as close as possible to the *top* of the condyle on a *line* running perpendicular through its length

The FLAIR images were saved as DICOM files. The signal intensity of the condylar marrow on each image was measured using public-domain software (ImageJ 1.37; NIH, Bethesda, MD, USA). The ROIs were placed over both the condylar marrow and gray matter (GM), which was taken as the reference point (Fig. 1). The size of the ROI and its position were determined according to the method of Yajima et al. [15]. A 5.9-mm^2^ ROI was placed in the temporal component, as close as possible to the mandibular condyle on a line running perpendicular to its length. A 4.5-mm^2^ ROI was also placed in the marrow at the top of the condyle.

The signal intensity ratio (SIR) of the condylar marrow was calculated using the signal intensity of the condylar marrow and GM (SIC and SIM, respectively) in the ROIs as follows: SIR = SIC/SIM [16].

The statistical analysis was performed using an unpaired *t* test to compare differences between the two groups. A correlation was also evaluated between the SIR and pain score obtained using the VAS with Pearson’s product-moment correlation coefficient. A *p* value of <0.05 was considered statistically significant.

## Results

No significant difference in the average patient age was seen between the 41 painful and 44 nonpainful joints (*p* = 0.77).

**Fig. 2:**
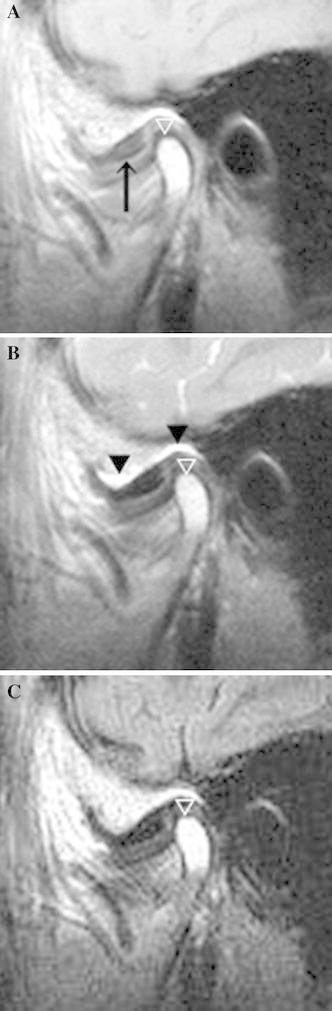
Painful TMJ with disk displacement and no reduction in a 68-year-old woman. A disk (*arrow*) was anteriorly displaced on a proton density-weighted image (**a**), and a large amount of joint effusion (*arrowhead*) was observed in the upper joint space on a T2-weighted image (**b**). The SIR was 1.90 for the bone marrow of the mandibular condyle on a FLAIR image (**c**). No bone marrow change was apparent on any MR images (*white arrow*) (**a**–**c**)

**Fig. 3:**
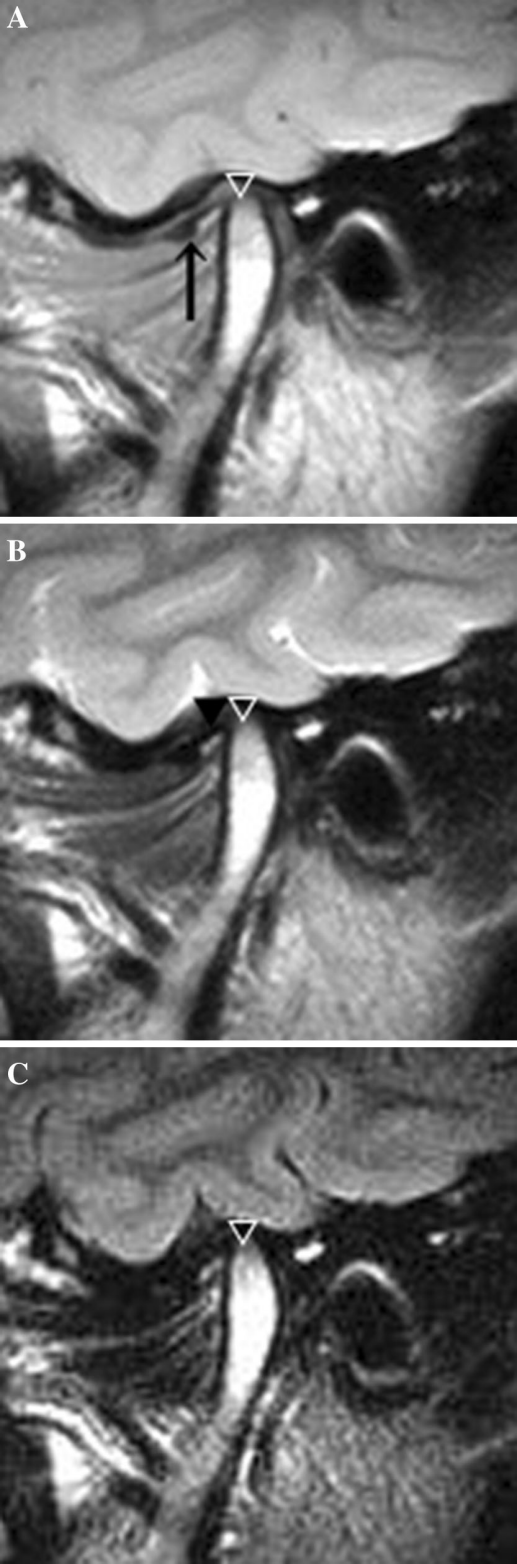
Nonpainful TMJ with disk displacement and no reduction in a 25-year-old woman. A disk (*arrow*) was medially displaced on a proton density-weighted image (**a**), and joint effusion (*arrowhead*) was observed in the lower joint space on a T2-weighted image (**b**). The SIR was 0.33 for the bone marrow of the mandibular condyle on a FLAIR image (**c**). No bone marrow change was apparent on any MR images (*white arrow*) (**a**–**c**)

A significant difference in the mean SIR was seen between the painful and nonpainful joints (*p* = 0.012) (Table 2). The mean SIR on the FLAIR images was 1.77 in the painful joints (Fig. 2) and 1.45 in the nonpainful joints (Fig. 3). However, the correlation coefficient was 0.12 (*p* = 0.0004), indicating no correlation between the SIR and pain score (Table 3).

**Table 2:** Difference in signal intensity ratio between painful and nonpainful joints

	No. of joints	Signal intensity ratio	
		Mean	SD
Painful joints	41	1.77	0.59
Nonpainful joints	44	1.45	0.57

*SD* standard deviation

**Table 3:** Ranges of signal intensity ratio and pain score

	Min	Max
Signal intensity ratio	0.73	1.65
Pain	0	68

## Discussion

The patient’s age should not be ignored when evaluating the bone marrow MRI signal intensity. Although the bone marrow is generally hematopoietic and red in youth, it gradually becomes fatty and yellow during development. The signal intensity of the bone marrow in the mandibular condyle thus changes starting after 10 years of age and continuing until age 25 [17]. In T1-weighted, proton density-weighted images, the signal intensity of the bone marrow changes from low to high in accordance with the proportion of its fat content. The signal intensity of the bone marrow is also influenced by the amount of fat without fat saturation on FLAIR images. Because there was no significant difference in the average age between the painful and nonpainful groups in this study, age differences cannot be considered as affecting the signal intensity changes in the mandibular condyle on FLAIR images.

In this study, the mean SIR on FLAIR images of painful joints was significantly higher than that on FLAIR images of nonpainful joints, indicating a difference in the condition of the bone marrow between the two groups. A correlation between increased protein levels and high signals on FLAIR images has been observed [18]. Therefore, higher signal intensity on FLAIR images may indicate increased protein levels in the condylar marrow in painful joints.

The joints in our patients had no visually apparent marrow change on proton density-weighted and T2-weighted images and appeared as normal bone marrow on MRI examination. According to Larheim et al. [8], bone marrow abnormalities appear to begin as edema and later change to osteonecrosis. Larheim et al. [8] histologically classified “the early stage of edema,” which is characterized by edematous fluid with serum protein exudate within the marrow interstitium, preservation of hematopoietic marrow elements, and no evidence of reticulin fibrosis. These serum proteins in the edema fluid include various proteins, such as albumin, alpha globulin, beta globulin, gamma globulin, fibrinogen, and prothrombin. In the early stage of edema, the mandibular condyle is considered to have protein-rich marrow. The condition of the painful joints in our study would fall into the very “early stage of edema,” and the increased signal of the condylar marrow on FLAIR images could reflect the elevated protein levels that occur at that stage.

Yajima et al. [15] studied the correlation between pain and the signal intensity of mandibular bone marrow on proton density-weighted images in TMJs with osteoarthritis. They reported an increase in proton density-weighted signals in TMJs with symptomatic osteoarthritis and suggested that this might reflect the early stage of edema as indicated by Larheim et al. [8]. Although joints with arthritis were not analyzed in the present study, our results are similar to those of Yajima et al. [15].

Osteonecrosis and bone marrow edema reportedly show abnormal signal intensity in the bone marrow [8]. Some authors have suggested that these changes in addition to joint effusion are possible factors in the etiology of TMJ pain [9, 11]. It has also been reported that blockage of the osseous microcirculation with associated intramedullary stasis may lead to severe and prolonged ischemia that eventually results in bone necrosis [19]. Osteonecrosis seems to be preceded by bone marrow edema, showing that venous stasis may lead to increased intramedullary pressure and may assume a role in ischemic damage and subsequent necrosis [20, 21]. The establishment of ischemia induces a vicious cycle involving cellular edema, increased pressure, and metabolic imbalance leading to cellular necrosis [19, 22]. Therefore, it is thought that ischemia may precede bone marrow edema and osteonecrosis in the condyle.

Ischemia is one cause of pain [23], and as soon as blood flow is inhibited, severe and localized pain will usually occur. Large amounts of lactic acid are produced by the anaerobic metabolism associated with this process, and a number of chemical substances such as bradykinin and proteolytic enzymes are produced because of cell damage in the affected tissue. These substances innervate the nerve endings that detect pain. Such pathological changes in the condylar marrow could cause an increase in the protein content of the marrow.

Although the pathologic processes that result in ischemia are poorly understood, and although it is difficult to reflect such changes at the cellular level, we suspect that ischemic change might be one factor involved in the development of TMJ pain.

No significant correlation was seen between the SIR and pain score, although a significant difference in the mean SIR was observed between painful and nonpainful joints. This was probably because of our exclusion of joints with visually apparent MR images. We suspect that a significant correlation would have been found between the SIR and pain score if a substantial number of joints with visually apparent changes had been studied. Further study is needed on this matter.

Pain in the TMJ is a symptom of TMJ disorders [24, 25]. A number of studies have investigated the causes of TMJ pain, including impingement and compression of the retrodiscal tissue [26, 27], inflammatory change in the retrodiscal tissue [28, 29], inflammatory change in the joint space resulting in joint effusion [30], capsulitis [31, 32], osteoarthritis [33–39], and alteration in the condylar bone marrow of the mandible [9, 11, 15]. Sano et al. [9] showed an association between a marked increase in pain and alteration in the bone marrow of the mandibular condyle. In another study by the same group [12], joints with marrow edema in particular showed a higher degree of pain than did those with osteonecrosis. An association between joint pain and pathological change in the bone marrow of the mandibular condyle resulting in a higher signal intensity on FLAIR images was also observed in the present study, supporting the results of the above-mentioned earlier study [9]. However, this does not mean that alteration in the bone marrow of the mandibular condyle causes pain. Only VAS scores were used to evaluate pain in the closed-mouth position in this study, so no detailed pain profile was available for the analysis. Therefore, the cause of TMJ pain might well be an abnormality other than bone marrow change. Further study is needed to determine the precise cause of pain, taking into consideration not only the rates, but also the various types of TMJ pain.

In conclusion, the present results suggest an association between abnormalities in the marrow of the mandibular condyle and pain. They also indicate that FLAIR is a useful tool in the clinical diagnosis of painful TMJs and offers a significant advantage over proton density- or T2-weighted images.
